# ‘It is just a prediction; it’s, like, not fact’: youth attitudes towards risk prediction tools and personalised preventive interventions for depression and anxiety

**DOI:** 10.1136/bmjment-2025-302327

**Published:** 2026-05-06

**Authors:** Nina Higson-Sweeney, Anna Peycheva, Josefien J. F. Breedvelt

**Affiliations:** 1 Institute of Psychiatry, Psychology & Neuroscience, King’s College London, London, UK; 2 Department of Experimental Psychology, University of Oxford, Oxford, UK; 3 Institute of Psychiatry, Psychology & Neuroscience, Amsterdam UMC Locatie AMC, Amsterdam, The Netherlands; 4 Division of Psychiatry, University College London, London, UK

**Keywords:** Depressive Disorder, Anxiety Disorders, Adolescent, Psychiatry

## Abstract

**Background:**

Risk prediction tools may help identify youth who are at risk of developing depression or anxiety and allow personalised preventive interventions to be delivered. However, with concerns for implementation, it is important to directly engage with youth to understand their attitudes.

**Objective:**

To qualitatively explore UK-based youth attitudes towards risk prediction tools and personalised preventive interventions for depression and anxiety.

**Methods:**

Online semistructured interviews were conducted with a convenience sample of youth aged 16–25 years (n=25) and analysed using reflexive thematic analysis.

**Findings:**

Analysis generated four themes: (1) *Helpful or harmful: risk prediction as a ‘double-ended sword’* explores the potential benefits and consequences of risk scores; (2) ‘*Taken with a grain of salt’: are risk prediction models the way forward?* focuses on participants’ scepticism towards risk prediction tools, including ethics and privacy; (3) ‘*It really depends on the person’: respecting the individual in prevention* emphasises the importance of personal choice and individual differences and (4) ‘*You still need like a person in the process’: the importance of human involvement* encapsulates participants’ belief regarding human involvement in development and implementation.

**Conclusions:**

While youth appear open to risk prediction tools and personalised preventive interventions, they highlighted concerns that must be addressed before implementation, including ethics, accuracy, privacy and feasibility.

**Clinical implications:**

Going forward, researchers should prioritise stakeholder involvement, using active collaboration to identify ways in which these concerns can be addressed, which may increase acceptability and uptake. Human contact, alongside agency and choice, are further factors to consider.

**WHAT IS ALREADY KNOWN ON THIS TOPIC**

- Risk prediction tools are increasingly used in physical healthcare, and several models have been developed to predict first-onset depression and anxiety, which typically occur before the age of 25. Previous research has raised concerns about the ethical, psychological and practical implications of using risk prediction in preventive psychiatry, especially with adolescents and young adults. Yet, little is known about youth attitudes towards the use of these tools in practice.

**WHAT THIS STUDY ADDS**

- This qualitative study explored UK-based youth (ages 16–25) attitudes towards risk prediction modelling tools and subsequent provision of personalised preventive interventions for depression and anxiety. Participants identified that risk prediction tools could lead to positive outcomes, including self-awareness, validation and increased help-seeking. However, prior to considering implementation, risk prediction tools must be refined with specific consideration for the ethics of their use, data privacy, model accuracy, availability of support, personal agency over involvement and human participation in the process.

**HOW THIS STUDY MIGHT AFFECT RESEARCH, PRACTICE OR POLICY**

- Given the shift in healthcare systems towards embedding prediction and prevention in practice, the identification of concerns that need to be addressed before youth are receptive to the implementation of risk prediction tools represents vital knowledge. We recommend that youth are actively involved in the development of risk prediction tools to increase acceptability and address concerns that may lead to low engagement or adverse effects.

## Background

 Common mental health conditions, including depression and anxiety, are among the top 10 contributors to the global burden of disease in youth.^1 2^ Recent prevalence estimates indicate that for youth aged 15–24 years, approximately 14% have an anxiety disorder and 2%–4% have a depressive disorder; this is in stark contrast to other mental health conditions such as bipolar (approximately 0.6%–0.7%) and schizophrenia (0.1%–0.2%), which have much lower prevalence.^1^ Not only do these mental health conditions have the potential to impact the attainment of developmental milestones but experiencing them in early adolescence and young adulthood is consistently predictive of long-term detrimental outcomes, including increased risk of recurrence, suicidal intent, reduced social functioning and poor physical health.^3^,^5^ In response, researchers and healthcare providers are increasingly exploring the utility of risk prediction tools to prospectively identify youth at-risk for developing these conditions, with the intention of delivering subsequent tailored preventive interventions that could avert a lifetime of poor mental health.

Risk prediction refers to the process of using an individual’s data to predict the likelihood of developing a specific condition over a set period and is commonly used in primary care settings to detect an individual’s risk of developing a physical health condition, like cardiovascular disease.^6^ The data these models are based on varies, but can include longitudinal cohorts, health records and surveys, drawing on data such as sociodemographic factors (eg, age and ethnicity), clinical information (eg, medical history and comorbidities) and contextual factors (eg, family history and environment). Current literature has developed several risk prediction models for the first onset of depression and anxiety in adolescence and young adulthood, though further development relating to performance, generalisability and data requirements would be needed in order to enable translation into practice.^7^,^12^ The potential benefits of using risk prediction within mental healthcare are widespread, including earlier identification and support, more targeted use of limited resources, supported clinical decision-making and improved service planning. An example of this is the development and delivery of personalised preventive interventions, which are tailored strategies designed to support an individual based on their predicted risk level (eg, 1:1 support for individuals at high risk, general wellness tips for individuals at low risk).^13 14^ As such, increased research efforts and funding are being directed towards this endeavour.

Given the surge in the development of risk prediction tools, concerns regarding implementation have been raised. These include logistical concerns such as feasibility and accuracy, ethical concerns such as informed consent, confidentiality and accurate comprehension, and broader concerns relating to stigma, public willingness to be informed about risk and the psychological burden that risk scores might bring.^15^,^17^ Findings have, however, been mixed, with further studies in the context of depression demonstrating that participants appreciated the increased self-awareness^18^ and welcomed the knowledge of their risk in order to make lifestyle changes.^19^ Consistently, calls for youth involvement regarding prediction model development and evaluation have been made to enable uptake and pre-identify future barriers to implementation.^15^ However, to date, no such study has specifically explored the views of youth in the UK using semistructured interviews, resulting in a lack of in-depth knowledge regarding whether these tools and interventions are wanted and what concerns may prevent engagement.

As such, this qualitative study aimed to explore how UK-based youth perceive risk prediction tools and the subsequent personalised preventive interventions that might be received for depression and anxiety.

## Methods

### Participants and recruitment

Participants were eligible to take part if they were (1) aged 16–25 years old, (2) currently living in the UK and (3) able to access the internet or travel to King’s College London for an interview. The study was not suitable for youth currently experiencing acute levels of mental health distress, including suicidal thoughts and/or self-harm, as assessed by a preparticipation screening questionnaire, which was developed by the research team to check that participants met the eligibility criteria. As this was a prevention-focused study, lived experience or diagnosis of a mental health condition was not part of eligibility; however, this information was collected to contextualise the sample, as prior experiences of mental health conditions and help-seeking can influence attitudes towards risk prediction and perceived risk.^17^ For example, positive experiences of help-seeking may increase trust, making participants more open to the idea of structured tools to support care. Alternatively, previous negative experiences may make participants more attuned to potential biases within models, with concerns relating to stigma and self-fulfilling prophecies.

Recruitment and data collection took place between September and November 2024. Participants were predominantly recruited through social media (ie, X, Instagram and LinkedIn), with the research team also emailing professional contacts and relevant organisations to distribute the study advert, such as Young Person Advisory Groups (YPAGs). Recruitment initially involved convenience sampling but switched to incorporate purposive sampling in October 2024 to increase sample diversity. Purposive sampling involved paying <£15 for the study advert to be boosted on Instagram for 5 days, targeting men aged 18–25 years old.

Recruitment was guided by information power^20^ and subsequently stopped once the research team determined that enough data had been collected to sufficiently answer the research question. Based on previous qualitative studies in this area, we anticipated 15–30 participants (see Figure S1 in online supplemental file 1).^21 22^ Once we had recruited 15 participants, the first author and senior author (NH-S and JB) examined the data with consideration to the five facets of information power, namely (1) the broad study aim, (2) sparse specificity of the sample, (3) growing theories in relation to risk prediction modelling, (4) mixed quality of dialogue and (5) cross-case analysis. Based on this, recruitment and checking of data continued until the quality of the dialogue had improved; this was determined by reading through interview transcripts, which were longer and included further follow-up questions that elicited deeper meaning and insight. It was subsequently agreed by NH-S and JB that information power had been achieved, and recruitment was closed.

### Procedure

The study was conducted in line with the Consolidated Criteria for Reporting Qualitative Research (COREQ) checklist^23^ (see Table S1 in supplementary materials). After registering their interest to participate through an online Qualtrics survey, participants took part in an online semistructured interview via Microsoft Teams. In-person interviews at King’s College London were also available but were not chosen. Interviews were delivered on a one-to-one basis by one of four undergraduate/postgraduate research assistants (including the middle author, AP), who ranged in experience with qualitative methods and received training and supervision throughout the project. Interviews were video-recorded and transcribed using Microsoft Teams’ inbuilt functioning, with all participants required to turn on their cameras for at least the start of the interview to verify their eligibility and prevent fraud. Interviews lasted an average of 22 min (SD=7.48; range=10–46) and were guided by a semistructured topic guide developed by the research team, with input from a YPAG (see online supplemental file 1). The YPAG was comprised of six young people aged 18–25 years old, who shared their honest feedback about the study and associated materials as part of a broader workshop in relation to the senior author’s (JB) fellowship. The topic guide asked questions relating to participants’ understanding, awareness and concerns of risk prediction tools and their understanding and attitudes towards personalised preventive interventions. An explanation of risk prediction tools and personalised preventive interventions was provided to aid any uncertainty from participants (see table 1), which were developed based on examples within the literature.^7^,^10^ Two vignettes were also included, as these can be helpful for eliciting participants’ attitudes towards a topic that they might be unfamiliar with or could be sensitive/controversial.^24^ These vignettes can be found in the online supplemental file 1. Participants received a £25 gift voucher as reimbursement and were given the option of choosing the pseudonyms used in place of their real names. Generated transcripts were checked for accuracy and anonymised by a research assistant, who also applied the pseudonyms.

**Table 1:** Key definitions provided to participants

Key term	Definition in topic guide
Risk prediction tools	Risk prediction tools are technologies that analyse data from various sources to estimate an individual’s likelihood of developing mental health conditions like depression or anxiety. These tools can use information from medical records, questionnaires and passive data collection from your phone and to make these predictions.
Personalised preventive interventions	Personalised preventive interventions are tailored strategies designed to help individuals based on their predicted risk levels. For example, someone identified as high risk might then receive a targeted preventive intervention, which consists of more intensive support such as 1:1 or group-based interventions or personalised coping strategies, while someone at low risk might receive general wellness tips and resources to maintain their mental health or no support at all.

### Data analysis

Data were analysed using reflexive thematic analysis and guided by Braun and Clarke’s six-phase approach.^25^ In phase I, the first author (NH-S) read and re-read transcripts while listening to interview recordings for familiarisation while making initial notes of interest. The senior author (JB) also engaged in familiarisation with three random transcripts, which were discussed during supervision. In phase II, NH-S undertook line-by-line coding using NVivo software at both a semantic (ie, participant-driven) and latent (ie, researcher-driven) level, meeting with JB to discuss progress and developing areas of interest. During phase III, NH-S integrated codes around central organising concepts that addressed the research questions, thus generating initial themes. Phases IV and V were iterative, with NH-S continually reviewing and refining themes with feedback from JB until themes were finalised. In the final phase, NH-S wrote up the findings with verbal and written feedback from co-authors.

Analysis was inductive and underpinned by the epistemological framework of critical realism, with the perspective that, although we cannot directly access participants' lived realities, interpretation of the available data allows us to gain insight into the phenomena under study and deepen our understanding of underlying structures driving attitudes and behaviours.^26^ Reflexivity, referring to the ongoing practice of researchers actively examining how their positionality influences the research process, was a core part of the analytic process.^27^ The research team engaged with reflexivity as part of group meetings, with NH-S also keeping a reflexive diary throughout data collection and analysis, focusing on how her prior experiences and understanding (eg, PhD in youth depression, family medical history used to predict physical health) influenced the production of knowledge. Further information on reflexivity can be found in online supplemental file 1.

## Results

25 participants were interviewed and included in the analysis (see tables2  3 for details).

**Table 2:** Participant demographics

Demographic	N
Age	21.04 (2.78)
Gender	
Female	15 (60%)
Male	9 (36%)
Genderfluid	1 (4%)
Transgender/gender diverse	
Yes	2 (8%)
No	17 (68%)
Prefer not to say	1 (4%)
Not collected*	5 (20%)
Sexual orientation	
Bisexual	8 (32%)
Gay/lesbian	2 (8%)
Heterosexual	12 (48%)
Queer	2 (8%)
Prefer not to say	1 (4%)
Ethnicity	
Asian	7 (28%)
Black African	1 (4%)
Mixed	1 (4%)
White British	13 (52%)
White Other	3 (12%)

Note. Age is mean (SD). Gender, transgender/gender diverse, sexual orientation and ethnicity are count (percentage). Age ranged from 16 to 25.

* This data was not collected due to an initial error in the Qualtrics survey, which was subsequently rectified.

**Table 3:** Participant pseudonyms and contextual characteristics

Pseudonym	Experience of a mental health condition	Experience of help-seeking*	Experience of risk prediction tools
Alice	Yes (Avoidant/restrictive food intake disorder, bipolar and depression)	Yes (primary, secondary and other)	No
Andres	No	No	No
Arif	Yes (anxiety)	No	No
Charlie	Yes (complex PTSD)	Yes (other)	Unsure
Chloe	No	No	No
Deepa	No	Yes (primary)	Unsure
Elijah	No	No	No
Hazel	Yes (anxiety)	Yes (other)	Unsure
Imogen	Yes (anxiety)	Yes (other)	No
Jacob	No	No	No
Jessica	No	No	No
Kabir	Yes (depression)	Yes (primary)	Yes
Kalani	Yes (social anxiety)	Yes (other)	No
Lucy	Yes (anxiety and PTSD)	Yes (primary and other)	No
Max	Yes (anxiety and depression)	Yes (primary and other)	Unsure
Mei	No	No	No
Myles	No	No	Yes
Noah	Yes (OCD)	Yes (other)	Unsure
Phoebe	Yes (depression)	Yes (other)	Yes
Poppy	Yes (anxiety and depression)	Yes (primary and other)	No
Robyn	Yes (anxiety, depression and disordered eating)	Yes (primary, secondary and other)	Yes
Roshni	No	Yes (other)	Unsure
Tara	Yes (depression and OCD)	Yes (other)	Unsure
Theodore	No	No	No
Violet	No	No	Unsure

* To preserve anonymity, participant responses have been altered to refer to primary care services (eg, general practitioners, National Health Service talking therapies), secondary care services (eg, Crisis Teams, Community Mental Health Teams and inpatient units), tertiary care services (eg, secure units) and other services (eg, charity helplines, university services and private care).

OCD, obsessive compulsive disorder; PTSD, post-traumatic stress disorder.

**Figure 1:**
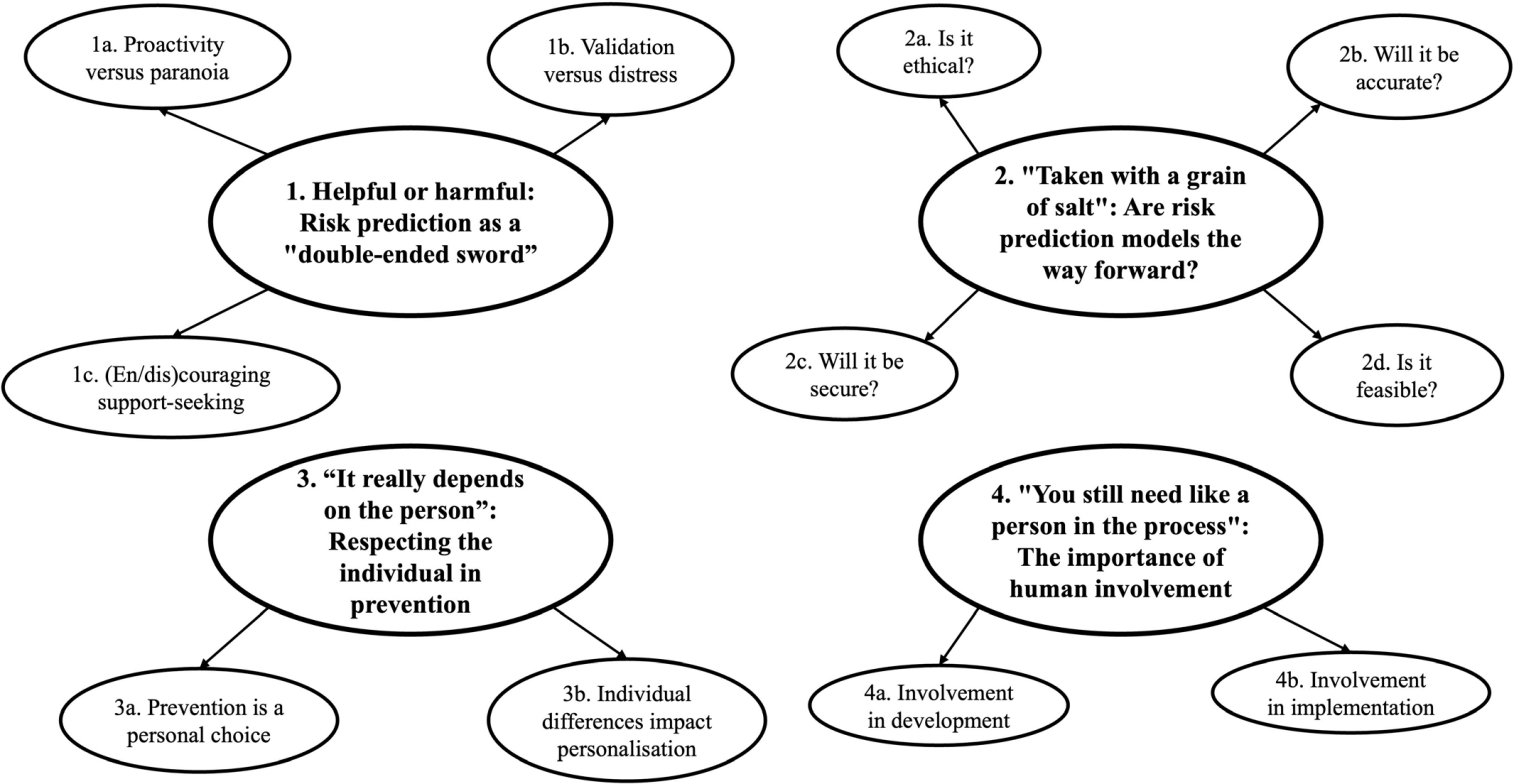
Thematic map.

Analysis generated four main themes that collectively capture youth attitudes towards risk prediction tools and resultant personalised preventive interventions (see figure 1 for a thematic map and Table S2 in online supplemental file 1 for additional quotes).

### Theme 1. Helpful or harmful: risk prediction as a ‘double-ended sword’

Participants viewed risk prediction models and subsequent risk scores as double-sided, with the potential for positive and negative outcomes for youth. These contrasting outcomes are explored through three subthemes.

#### 1a. Proactivity versus paranoia

In the context of being identified as at high risk for developing a mental health condition, most participants discussed the benefit of heightened self-awareness, particularly for those who are unaware of their potential risk. They explained how the resultant proactivity from self-awareness could lead to effective prevention.

(Risk scores) might be really helpful because you can just kind of keep an eye out before you feel the need to go and talk to the GP (…) it could be really helpful as a kind of preventative, look out for this type of thing. (Robyn)

However, participants questioned whether this awareness could lead to hyper-vigilance and paranoia, with high-risk scores prompting youth to overanalyse and misinterpret their behaviours as symptoms. Some suggested this could result in youth *placebo-ing* (Robyn) themselves and creating a self-fulfilling prophecy.

I suppose I'd be hyper-aware all the time of like what I'm doing and how my mental health is (…) which might be kind of a bit too much if I'm constantly thinking, is this going to affect my mental health? (Hazel)

#### 1b. Validation versus distress

Participants perceived that receiving a risk score could be validating, particularly for those who might be experiencing early symptoms or have a family history of mental health conditions. Subsequently, being offered a preventive intervention could then assure youth that they are being taken seriously and that others care.

If you were already going through mental health issues when you got the assessment, I'd say it’s almost quite kind of affirming that, okay, yes, someone else recognises that this is what I'm going through. (Robyn)

In contrast, for youth identified as being at low risk, receiving this score could provide a sense of relief or security from knowing they are unlikely to develop a mental health condition. Participants thought this could increase confidence and help youth worry less when experiencing emotions like sadness or anxiety.

If I was identified as low risk, it would like take a weight off my shoulders a little bit and I would maybe feel like I could stretch myself like for example the sleep, the socialisation, I would maybe push myself (…) to like not worry about these things as much. (Poppy)

However, most participants expressed that being assigned a risk score could cause feelings of distress, overwhelm and uncertainty, particularly if identified as high risk or if the score did not match a youth’s experience. They were concerned that this could exacerbate any experienced symptoms and cause youth to question their lived realities and withdraw from life and those around them. In this way, risk scores could become *quite a heavy burden* (Lucy), making participants question the usefulness of telling youth their risk score.

#### 1c. (En/dis)couraging support-seeking

Participants thought that a clear benefit of being identified as high-risk was the prompting and subsequent access to follow-up support, which would negate some commonly experienced barriers to help-seeking, like long waiting times, and could result in effective prevention.

It'd be quite concerning to be labelled high risk, it’s still I think positive in a way because it’s trying to seek that positive support and trying to urge you to speak to someone (…) it’s kind of leading to seek out treatment and things like that, it’s aiming to deliver more positive mental health. (Elijah)

However, several participants emphasised that *low risk doesn't mean no risk* (Charlie) and were concerned that those at low risk would disregard their mental health or avoid help-seeking. Participants expressed the importance of communicating to anyone at low risk that they could still get access to help if needed and that they would be taken seriously.

If I was going through a particularly bad time and I was being labelled as low-risk, I think the issue would then be that I would feel more encouraged to, like, dismiss what I'm feeling. (Andres)

### Theme 2. ‘Taken with a grain of salt’: are risk prediction models the way forward?

While participants were generally receptive to the idea of risk prediction models and personalised support, the majority voiced their scepticism for at least one area of concern. These concerns are explored through four subthemes.

#### 2a. Is it ethical?

Some participants queried the ethics of using risk prediction models to assign a youth with a risk score or label. Despite increased awareness, mental health is still a stigmatised topic, and participants emphasised that discussions of risk should be approached cautiously. They questioned the impact of assigning a high-risk score to someone unaware of potential risk or giving a low-risk score to someone already struggling, in case this worsened their mental health.

Being labelled as being at risk of having a mental health condition kind of puts like a stigma on them, especially if they weren't thinking (…) about it before. So if for example like they would be like surprised to receive the at risk like status of having mental health condition I think if it’s not done in a sensitive way then it could put people off. (Andres)

Many participants expressed that risk prediction models seemed deterministic and reductionist, which contradicted their understanding of mental health being approached holistically. Participants challenged the appropriateness of an algorithm to condense something as multifaceted as mental health into a single score, particularly if that determined subsequent support.

I think it can be quite deterministic (…) you have a 90% chance of developing depression. It’s like, oh, yeah? Well, that’s pretty sure it’s going to happen. (Phoebe)

Participants emphasised the importance of transparency when using risk prediction tools to ensure that youth are fully informed of how risk scores are calculated and what they mean. They also asserted that risk scores should be *carefully phrased* (Phoebe) and communicated in a sensitive, discrete way to not provoke additional distress or harm.

#### 2b. Will it be accurate?

Participants understood that many factors could contribute to a youth’s risk of developing a mental health condition, including biological and environmental components. They doubted that a risk prediction tool could capture all necessary factors, including subjective reactions to adversity and fluctuations over time, which would hinder the accuracy of a risk score.

(Algorithms) do have to always, in my opinion, be taken with a grain of salt. You can't use these algorithms to inherently always 100% predict human behaviour, because like we're not computers. (Charlie)

Some participants highlighted that the accuracy of a model would depend on the reliability of its data, which could be variable. Data like medical records and physiological measures were perceived as more accurate than self-report questionnaires. Nevertheless, participants recommended that, in order to build the most comprehensive picture possible, many different sources of data, including physical health, should be used to inform risk and that risk scores should be updated periodically to capture change. Participants were hopeful that continued research would improve the accuracy of models.

Your medical records are things that can't be faked (…) and it’s usually up to date also a lot of the time, so if you're taking it off the medical records then you can trust it. (Roshni)

#### 2c. Will it be secure?

Almost all participants shared concerns related to data privacy, protection and ownership. As the data used to calculate risk prediction scores is sensitive and not necessarily anonymous, participants were apprehensive about where and how data would be stored, who would have access to it, and what the consequences of others learning this information could be.

It is also quite concerning because who’s actually going to be in control of that data, you can't really guarantee that it’s going to end up in the right hands (…) if it gets in the hands of employers and they get decide they don't want to take someone on their workforce because they might be at risk by missing too many days off (…) it could get quite dangerous, unless there’s some very tight laws put in place to protect people. (Violet)

Some data was perceived as more acceptable than others, such as medical records rather than data collected through mobile phones. Interestingly, data that participants were less comfortable with being collected, such as mobile phone data, tended to be data that they perceived as providing more accurate indications of risk, highlighting a tension between privacy and accuracy.

My phone probably has a greater knowledge about those things that I was saying about human factors etcetera that a medical professional would not see, so perhaps that would make it more accurate and I should trust in it, but I don't know how comfortable I would feel with that. (Poppy)

Similar to ethical concerns, participants were keen to see open and honest communication with youth regarding the data that risk prediction models would use, as well as fully informed consent for its collection, use and withdrawal.

#### 2d. Is it feasible?

Participants recognised the usefulness of risk prediction tools in the UK context, where demand for mental health support currently outstrips supply. Participants felt like risk prediction models could make the identification and referral process *quite smooth* and *streamlined* (Andres), with early intervention potentially preventing the development of more severe—and costly—mental health conditions. In ideal circumstances, risk prediction models would allow youth to receive the most appropriate care.

I think it makes sense in terms of preventative things like, trying to get in there before it turns into a bigger issue. And just cost-wise, we can't provide like the highest level of care for everyone. (Lucy)

However, not all participants were convinced that widespread implementation of risk prediction models and personalised preventive interventions is feasible, particularly in relation to finances, logistics and infrastructure. They also questioned the ethics of only prioritising those at high risk, especially in relation to doubts of accuracy, and whether those most in need would be the ones to receive the support.

Do we have the infrastructure to do that? (…) logistically and financially as well to, you know, offer counselling session to everyone who is at high risk, you know, especially one-to-one sessions, that’s going to be very expensive. (Phoebe)

### Theme 3. ‘It really depends on the person’: respecting the individual in prevention

Participants emphasised the importance of the individual in the context of risk prediction models and preventive interventions from two angles: personal choice and individual differences.

#### 3a. Prevention is a personal choice

Participants were clear that engaging with risk prediction models and interventions needs to be a personal choice. Not every youth will want support following a risk score, and most participants felt this choice should be respected and not *forced down my throat* (Theodore), even for those at high risk, although some did question this.

I don't think you should be pushed through a system you don't believe you'd benefit from just because an algorithm said you should. (Violet)

Ensuring that youth have agency over their involvement with risk prediction and preventive interventions was paramount to participants. They emphasised that youth should be in control of what information and treatments they receive and how they receive them because *a lot of the time in mental health support, autonomy is taken away* (Charlie). Participants were clear that interventions should be opt-in and delivered at the individual’s own pace, although issues regarding denial and lack of motivation were recognised.

Getting your result from the prediction model but then being able to kind of choose the level of support even though a certain level of support is recommended. (Alice)

#### 3b. Individual differences impact personalisation

Participants were clear that one size does not fit all for preventive interventions and that personal preferences need to be considered. This was well-exemplified by participants’ varying preferences for intervention, including one-to-one counselling, group therapy, mental health apps and self-help resources. Across the sample, there was no consensus on what format personalised preventive interventions should take.

Participants believed that personalisation would make preventive interventions more effective, as they would be tailored to the individual’s needs and informed by their data. This was contrasted against the current National Health Service (NHS) approach, where *everyone is treated the same way, which isn’t a good thing* (Jacob). However, they also explained that the usefulness of risk prediction and prevention would likely differ case-by-case, regardless of personalisation, because it would not necessarily provide the type of support youth are looking for.

Interventions don't work for everyone, so it would be difficult to say (…) everyone should do this one therapy and it’s going to work for you because people are different and they respond to different ways of learning (…) personalisation has to come into it in some way for it to be effective. (Jessica)

### Theme 4. ‘You still need like a person in the process’: the importance of human involvement

Ultimately, participants stressed the need for human involvement with risk prediction models and personalised preventive interventions, both in relation to development and implementation.

#### 4a. Involvement in development

Some participants stressed the importance of involving stakeholders in the development of risk prediction models and personalised interventions. Stakeholders could be youth but also important individuals around them; the focus was more on ensuring human oversight and input.

Just engaging with people with the creation of these models to make working sure that people are interested in that sort of thing and also yeah, making sure people are happy with how the programmes are and the kind of data they're collecting. (Mei)

A key part of involvement for participants was educating youth about these tools and the importance of prevention, potentially due to their own uncertainty. Education and a clear understanding of what these tools are, how they are created, what data is used, how the risk score is determined, and the benefits of prevention could alleviate some of the concerns highlighted in previous themes and encourage engagement.

Discuss how they work, so people aren't like as intimidated about using these tools and (…) people feel like it’s accessible and it’s not looked down upon so a lot more people can get the benefits. (Chloe)

#### 4b. Involvement in implementation

Participants were clear that human involvement should continue across development and into delivery. While they were not opposed to an algorithm calculating a risk score, they wanted it to be confirmed by a healthcare professional who might detect the nuance of their individual situation.

I see those models as more of early identification or again risk identification, and then you take it further with the doctor or something. I wouldn't personally be confident with a risk prediction model fully diagnosing me. (Kabir)

Participants emphasised that options for personalised preventive interventions should be introduced by and discussed with trained healthcare professionals. They felt that having the *space to kind of talk through your options with somebody* (Mei) was important, giving youth the chance to build rapport with another human and be seen as a person rather than a number. Participants discussed how someone knowledgeable would be able to explain the pros and cons of each treatment and ensure that the best intervention was identified and properly provided. They were concerned about the impact of a youth receiving a label and being signposted to an intervention with no human follow-up.

Just being told, ‘we think you should go to therapy’ over an e-mail isn't probably the best way to go about it. (Robyn)

I'd rather like an actual like kind of person like explain and kind of help you to kind of maybe choose what’s right for you or see what can actually help you rather than just like a big chunk of text with like hyperlinks. (Arif)

However, personal choice was still emphasised, with the understanding that not everyone would want to discuss something so sensitive and private face-to-face.

## Discussion

The current study explored the attitudes of UK-based youth towards risk prediction tools and personalised preventive interventions for depression and anxiety. The four themes generated highlight that while this approach could lead to positive outcomes like self-awareness, validation and increased help-seeking, much work is still required before implementation, with concerns for ethics, accuracy, privacy, feasibility, agency and human involvement.

Our research offers nuance in light of initial advances in prediction modelling and personalised interventions such as just-in-time adaptive interventions, which are digital mental health interventions that deliver personalised support in real-time based on their immediate context.^7 8 28^ As the beneficiaries of such methodological and statistical advances, it is vital that the voices of youth are at the fore, and our participants emphasised stakeholder collaboration to ensure that these models accurately capture their contexts and experiences. Not only will their active involvement in the development and testing of these tools help to ensure that they are appropriate and effective, but understanding their attitudes towards risk prediction tools can give us insight into potential uptake and engagement and allow researchers to proactively address areas of concern. Learning can also be drawn from the digital mental health intervention field, where there is currently an increased focus on co-production and co-creation to resolve issues with adherence and acceptability.^29 30^

Our findings align with previous research that has qualitatively explored risk prediction and prevention, although this study is the first to use semistructured interviews to specifically explore the attitudes of UK-based youth in the context of depression and anxiety. Other studies to date that have explored stakeholder perspectives and global youth perspectives have expressed similar concerns to our participants, including accuracy, comprehension, feasibility, privacy and stigma.^16 21 22^ Ethical concerns were a particular issue, including unintended consequences following categorisation, such as the development of self-fulfilling prophecies, the exacerbation of distress and the avoidance of help-seeking. Participants highlighted the importance of educating youth on these models to help assuage some of these fears, alongside the need for transparency and collaboration to help build trust. However, our participants were also vocal about the positives of risk prediction and prevention, which has generally seen less attention in the literature. In a global qualitative study involving youth, parents, teachers and healthcare practitioners, Kohrt *et al*^16^ identified potential benefits of risk calculators for youth depression, including improved self-care and objectivity. Our participants also emphasised the potential benefit of self-awareness for proactive prevention but additionally spoke about the benefits of validation and follow-up access to support, highlighting the potential of these tools in addressing some common barriers to help-seeking, including fears of not being taken seriously and long waiting times.^31^ Our study suggests that while much work is needed before risk prediction tools can be implemented in practice, youth do recognise the potential benefits and utility of these tools. However, it is important to recognise that this was all discussed in the context of depression and anxiety; further qualitative research is needed to understand youth attitudes towards risk prediction tools for other mental health conditions, such as bipolar, eating disorders and psychosis.

Moreover, our study was the first to explore subsequent personalised preventive interventions based on risk scores. Youth highlighted the importance of follow-up support being available after receiving a score, with most expressing a preference for human contact and involvement, which is often reflected in the digital mental health literature.^32 33^ While youth recognised that not everyone would want subsequent support, they felt that tailoring an intervention to their data and individual circumstances would likely make prevention more effective and were positive about the prospect of this provision. This aligns with findings from a recent qualitative study on UK youth perspectives on technology-enabled personalised support for depression,^34^ alongside general discourse in the literature highlighting the importance of person-centred care within mental health decision-making. However, to date, there is limited provision and understanding of evidence-based preventive interventions,^35^ which participants were concerned about. Developing risk prediction tools is only half of the picture; further research is needed regarding subsequent effective prevention before anything is implemented. Population-based prognostic modelling, where groups at highest risk are identified, could be a helpful strategy to ensure sufficient evidence-based interventions are commissioned.^36^

While this study has numerous strengths, there are limitations. First, the sign-up survey had fraudulent participants, which was identified through IP addresses and mismatched information. Fraudulence was addressed through visual identity checks of participants at the beginning of interviews and re-watching interview recordings to assess the authenticity of responses. This resulted in one participant being withdrawn from the study, as their reliability could not be determined. Additionally, four interviews were <20 min and did not produce as much rich data as other interviews. However, it felt inappropriate to exclude this data based on a lack of depth, and recruitment continued until information power had been reached. Finally, for ease of understanding, risk prediction tools were introduced to participants as providing a risk score or label, despite this not being the only approach. Although we did not present risk scores as only being discrete (ie, high vs low), we found that participants tended to interpret the concept in this way and seemed to find this anchoring useful in helping to express their thoughts. Future research should ensure that stakeholder feedback is provided during the development of specific models for tailored information, including consideration for how the output of risk prediction tools is framed.

### Clinical implications

Prior to the implementation of risk prediction models in the prevention of depression and anxiety in adolescence and early adulthood, several concerns from youth need to be addressed. There remains a lack of clarity around the clinical applications of risk scores, in part because there are more models than actual tools ready for clinical use. Youth are concerned about ethics, accuracy, privacy, feasibility and agency, which all need to be incorporated in the future development of such tools, with youth actively involved in this process. These concerns and priorities from youth need to be considered in the context of personalised interventions, too, including the accommodation of individual differences and the need for human involvement. However, it is promising that youth see the benefit of these tools and seem receptive to the hypothetical idea of personalised preventive interventions, particularly given the NHS’s new 10-year Health Plan^37^ which seeks to make better use of AI and expand the structure of England’s healthcare system to incorporate prediction and prevention. However, if risk prediction tools and personalised preventive interventions are to be implemented within healthcare systems like the NHS, these services need to be set up to cope with an increased demand for support, including adequate resources and established care pathways. With further research into the development of these models that places co-production at the fore, these predictive tools and interventions could have an important place in future precision psychiatry and clinical practice and contribute to the transformation of the NHS into a more sustainable system.

## Supplementary material

10.1136/bmjment-2025-302327 online supplemental file 1

## Data Availability

No data are available.
