# Supplementary material for: ‘It is just a prediction; it’s, like, not fact’: youth attitudes towards risk prediction tools and personalised preventive interventions for depression and anxiety
Source: BMJ Ment Health. 2026 May 6;29(1):e302327. doi: 10.1136/bmjment-2025-302327 (PMC13150875; doi:10.1136/bmjment-2025-302327)
Supplement: online supplemental file 1 [file bmjment-29-1-s001.pdf]

Supplementary Materials

Interview Topic Guide

Introduction (5 minutes)

- **Purpose of the Interview:**
    - Explain the aim of the study.
    - "Thank you for agreeing to participate in this interview. The aim of this study is to understand how young people like yourself perceive the use of risk prediction tools for mental health, specifically for depression and anxiety. We are interested in learning about your attitudes towards these tools, and your views on receiving personalised preventive interventions. Your insights will help us understand how to make these tools and interventions more acceptable and effective for young people."
- **Reminders:**
    - Assure participants of confidentiality and anonymity (audio recordings will be transcribed, identifiable data will be removed and participants will be assigned pseudonyms).
    - Right to withdraw at any time during the interview without giving a reason.
    - Can ask for the interview to be paused or to have a break.
    - Do not have to answer any questions they do not want to.
    - Are you still happy to continue with the interview?
    - Do you have any questions before we begin?

[Begin recording]

Section 1: Introduction to Risk Prediction Tools and Personalised Interventions (5 minutes)

- What is your understanding when I say 'risk prediction tool'? What does that mean to you?
    - If I start using synonyms like 'early warning system', 'prediction modelling', 'prognostic tools', 'risk score' or 'risk stratification models', does your understanding change? If so, what is your current understanding?
- "To give you some context, **risk prediction tools** are technologies that analyse data from various sources to estimate an individual's likelihood of developing mental health conditions like depression or anxiety. These tools can use information from medical records, questionnaires, and passive data collection from your phone and to make these predictions. **Personalised preventive interventions** are tailored strategies designed to help individuals based on their predicted risk levels. For example, someone identified as high risk might then receive a targeted preventive intervention, which consists of more intensive support such as 1:1 or group-based interventions or

personalised coping strategies, while someone at low risk might receive general wellness tips and resources to maintain their mental health or no support at all.

  - During our discussion, we'll explore your thoughts and feelings about these concepts, any concerns you might have, and your views on how effective and acceptable these interventions might be for you and your peers."

Section 2: Vignette 1 - Risk Prediction Modelling (15-20 minutes)

- **Vignette Description:**
  - "Imagine a tool that collects data from various sources such as questionnaires, longitudinal studies, medical records, and your phone to actively predict analyses patterns and uses algorithms to identify individuals who are at high, medium, or low risk for these mental health conditions. I am now going to ask you some questions with this idea in mind."
- **Understanding and Awareness:**
  - Have you heard about tools like this before? What do you think about them?
- **General Attitudes:**
  - What are your initial thoughts or feelings about using such models to predict mental health risks?
  - Do you think these models could be useful/helpful? Why or why not?
- **Trust and Accuracy:**
  - How accurate do you think these models might be in predicting who is at risk for depression or anxiety?
  - How trustworthy do you find the technology and methods behind these predictions? What makes you say that?
- **Labelling and Categorisation:**
  - How would you feel about being identified as high or low risk for depression or anxiety?
  - What concerns, if any, would you have about this kind of labelling?
- **Impact on Personal Life:**
  - How do you think being labelled as high risk might affect your life (e.g., socially, academically, personally)?
  - How do you think being labelled as low risk might affect your life (e.g., socially, academically, personally)?
- **Other Concerns:**
  - Would you have any other concerns about these models and how they are used to identify you as high or low risk?
    - E.g., privacy concerns regarding data, stigma related to being identified as high or low risk

Section 3: Vignette 2 - Personalisation in Prevention (15-20 minutes)

- **Vignette Description:**
  - "Now imagine that based on your risk level, you receive tailored interventions to help prevent depression or anxiety. For example, if you are identified as

high risk, you might receive regular counselling sessions, access to mental health apps, or personalized coping strategies. If you are at low risk, you might receive general wellness tips and resources to maintain your mental health or no support at all. I'm going to ask you some more questions with this in mind."

- **Relevance and Acceptance:**
  - How do you feel about receiving preventive interventions based on your predicted risk level?
  - What types of interventions and level of personalisation would you find acceptable or helpful? What makes you say that?
- **Autonomy and Control:**
  - How do you feel about being offered these interventions? Would you prefer to have a choice in whether these interventions are offered to you (e.g., opt-in, consultation)?
  - What could be a suitable way of introducing these interventions to you?
    - ♣ What would help you feel more comfortable to receive these interventions?
- **Effectiveness and Impact:**
  - Do you think personalised interventions could be effective in preventing depression or anxiety? Why or why not?
  - How might these interventions positively or negatively impact your mental health and overall well-being?

Section 4: Recommendations and Final Thoughts (5 minutes)

- **Improvements and Suggestions:**
  - Based on what we've discussed today, are there any further improvements or changes you would want to see for risk prediction models and personalised interventions?
  - How can professionals better address your concerns regarding these tools?
- **Final Thoughts:**
  - Is there anything else you would like to add about your views on risk prediction models and personalised preventive interventions?
  - Do you have any questions for me about the study or the topics we discussed?

[Stop recording]

Closing (2-5 minutes)

- **Thank You:**
  - Thank the participant for their time and insights.
- **Next Steps:**

  - Briefly explain what will happen next in the study (e.g., analysis of data, potential follow-up).
  - Receiving a £25 Amazon voucher; double check that this is the email they want it sent to.
- **Contact Information:**
  - Provide contact information for any further questions or concerns.

This topic guide is designed to cover the main research questions while allowing for flexibility based on the participant's responses and the flow of the conversation.

Reflexivity Statement

Reflexivity is an ongoing process where researchers critically examine how their positionality, including their knowledge, experiences, beliefs, and attitudes, may influence the research process and subsequently impact the knowledge that is generated (Berger, 2015). Reflexivity is a core component of Reflexive Thematic Analysis (Braun & Clarke, 2021), with the afforded awareness allowing researchers to actively engage with their assumptions and develop a deeper understanding of the part they have played in constructing the data. In turn, this engagement and understanding can enhance research quality by increasing transparency, criticality, and overall credibility (Berger, 2015).

All three authors are women in their 20's and 30's who are active researchers in the field of young people's mental health. Below are individual reflexivity statements that consider some of the ways in which their positionality impacted the research.

NH-S

I became involved with this project towards the end of my PhD, which focused on depression in young people. As such, while the analysis was inductive and driven by the data provided by the participants, I had a pre-existing understanding of what depression looks like in this population and an expectation (based on the literature and previous research I have undertaken) of how young people might respond to the use of such a tool. Furthermore, although I have never actively used risk prediction tools, I am familiar with GPs using factors like my ethnicity and family history to highlight physical health conditions I might be at-risk of developing. Subsequently, I have some understanding about how it might feel to be determined at-risk for something that I was previously unaware of.

Keeping a reflexive diary throughout the process of analysis was key to maintaining awareness of how my prior experiences and knowledge might be impacting the analytic process. While it was not possibly to *switch off* my assumptions, this awareness did allow me to adjust some of my behaviours to try and account for my own bias. For example, I found myself drawn to data that aligned with my own experiences of being told I am at-risk and found the process of coding much more intuitive when participants raised concerns related to data privacy and protection, as these issues were similar to conversations I had previously had with young people. Through reflexivity, I was able to identify moments where I had paid greater attention to certain elements of transcripts and engage in additional coding to try and make sure that all relevant data was captured. It also helped me to identify areas to discuss in

meetings with the wider research team, whose different perspectives and experiences afforded new insights to the analysis and my approach to the data.

AB

My academic background in psychology (BSc) and global mental health (MSc) positions me with a perspective that values evidence-based and person-centred approaches to mental health. I have no direct experience of using risk prediction tools. However, I have worked as a research assistant on personalised prevention for youth depression and anxiety and may therefore hold an informed yet invested perspective on the potential of such approaches, which I recognise might shape my interpretation of participants' views. Having lived across multiple countries and experienced different healthcare systems, I am also mindful of how different cultural and structural contexts might influence young people's perception of and attitudes toward mental health, risk, and prevention.

JB

I conceptualised this study and brought together a team of researchers to collaborate on its development and implementation. I lead a university-based research group focused on understanding how to best design and deliver preventive approaches for adolescents and young adults, particularly within the UK educational, health, and social care systems. Although I do not have direct experience in using risk prediction tools, my research has centred on identifying risk and resilience factors that may support stratification to preventive interventions and personalisation. I have also been following the literature on the use and performance of risk prediction tools for prevention. My role as lead of a lived experience advisory group as part of my Prudence Trust Research Fellowship has been especially influential in shaping my perspective. Through ongoing discussions with group members, I have developed a more nuanced understanding of the benefits and risks that risk prediction tools may present, as well as the importance of grounding this research in young people's lived experiences.

Figure S1. Participant Flow Diagram

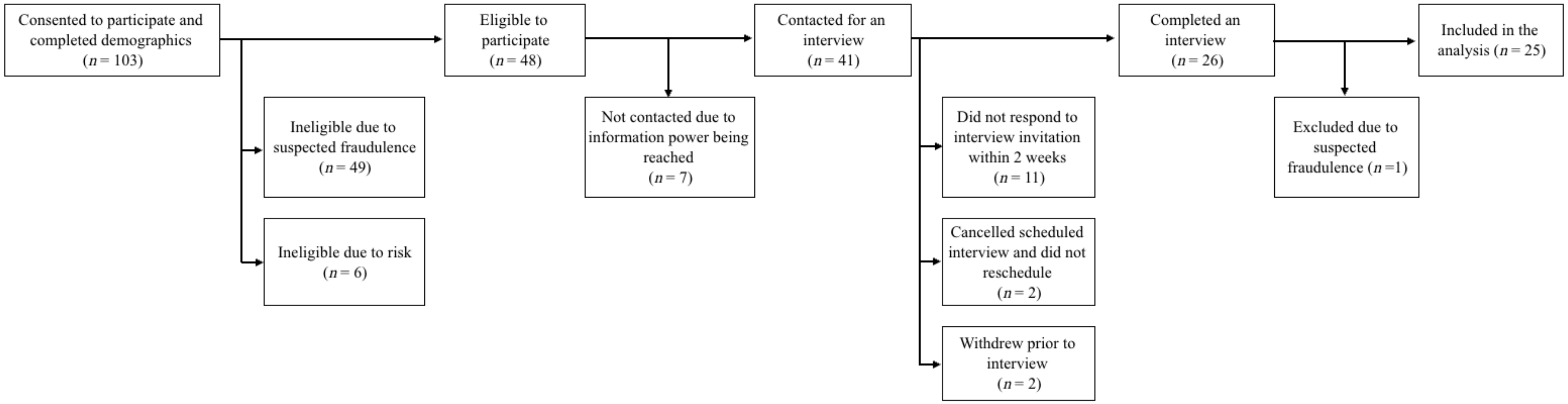

Table S1. COREQ Checklist (Tong et al., 2007)

No.	Item	Description	Response
**Domain 1: Research team and reflexivity**			
**1.**	**Interviewer/ facilitator**	Which author/s conducted the interview or focus group?	Interviews were conducted by AP and research assistants, with supervision from NH-S and JB; see *Methods>Procedure* and *Acknowledgements* for this information.
**2.**	**Credentials**	What were the researcher's credentials?	NH-S and JB both have PhDs, and AP has a MSc in Global Mental Health; see *Reflexivity Statement* in supplementary materials for further information.
**3.**	**Occupation**	What was their occupation at the time of the study?	NH-S was a lecturer and postdoctoral researcher, AP was a research assistant, and JB was a Prudence Trust Research Fellow; see *Reflexivity Statement* in supplementary materials for further information.
**4.**	**Gender**	Was the researcher male or female?	The whole research team identified as female; see *Reflexivity Statement* in supplementary materials for further information.
**5.**	**Experience and training**	What experience or training did the researcher have?	NH-S has had substantial training and experience in conducting qualitative studies, particularly when using semi-structured interviews and reflexive thematic analysis. NH-S provided training and supervision to the rest of the research team for this study, who are experts in the area of risk

			prediction modelling and development; see *Methods>Procedure* for further information.
**Relationship with participants**			
**6.**	**Relationship established**	Was a relationship established prior to study commencement?	Participants had contact with the research team via email prior to the interview being conducted, which provided them with an opportunity to know who they would be interviewed by and to ask any questions they may have had; see *Methods>Procedure* for an overview of the study procedure.
**7.**	**Participant knowledge of the interviewer**	What did the participants know about the researcher?	Information regarding who the lead researchers were and their goals for the study were provided in the Participant Information Sheet, which were presented to potential participants prior to consent via an online survey hosted on Qualtrics.
**8.**	**Interviewer characteristics**	What characteristics were reported about the interviewer/facilitator?	As above.
**Domain 2: Study design**			
**Theoretical framework**			
**9.**	**Methodological orientation and theory**	What methodological orientation was stated to underpin the study	This study was underpinned by a critical realist, contextualist perspective, which aligns well with the analytic method of reflexive thematic analysis; see *Methods>Data analysis* for a further explanation.
**Participant selection**			
**10.**	**Sampling**	How were participants selected?	Convenience sampling was initially used, before switching to purposive

			sampling halfway through recruitment in October 2024 to ensure greater diversity. Purposive sampling involved paying under £15 for the study advert to be boosted on Instagram for five days, targeting men aged 18 to 25 years old; see *Methods>Participants and recruitment* for further information.
**11.**	**Method of approach**	How were participants approached?	All participants consented and registered their interest in taking part in the study via an online Qualtrics survey; this was then followed up by a member of the research team via email.
**12.**	**Sample size**	How many participants were in the study?	Twenty-five participants were interviewed and included in the analysis; see *Results* for further information.
**13.**	**Non-participation**	How many people refused to participate or dropped out? Reasons?	Forty-one individuals were contacted for an interview; 11 did not respond to the interview invite, two cancelled the interview and did not reschedule, and two withdrew prior to the interview as they no longer had the availability. One participant was interviewed but later withdrawn by the research team as we were unable to verify that they were not fraudulent. See *Figure S1* in supplementary materials for further details.
**Setting**			

**14.**	**Setting of data collection**	Where was the data collected?	All interviews took place online via Microsoft Teams, although in-person interviews at King's College London were also offered.
**15.**	**Presence of non-participants**	Was anyone else present besides the participants and researchers?	No, all interviews were on a one-to-one basis.
**16.**	**Description of sample**	What are the important characteristics of the sample?	Important demographic characteristics related to the sample as reported in *Table 2* and *Table 3*. Most characteristics have been reported as aggregate to aid anonymity, with select characteristics associated with pseudonyms to provide context to their quotes.
**Data collection**			
**17.**	**Interview guide**	Were questions, prompts, guides provided by the authors? Was it pilot tested?	A semi-structured topic guide was developed to help guide the interviews and is available at the beginning of the *Supplementary Materials*. The research team practiced the interviews on one another prior to undertaking interviews with participants.
**18.**	**Repeat interviews**	Were repeat interviews carried out? If yes, how many?	No, each participant was only interviewed once.
**19.**	**Audio/visual recording**	Did the research use audio or visual recording to collect the data?	Audio and video recordings were collected for all interviews via Microsoft Teams.
**20.**	**Field notes**	Were field notes made during and/or after the	No.

		interview or focus group?	
**21.**	**Duration**	What was the duration of the interviews or focus group?	Interviews ranged from 10 to 46 minutes; see *Methods>Procedure* for further information.
**22.**	**Data saturation**	Was data saturation discussed?	Data saturation is an outdated approach within qualitative research and is underpinned by essentialist assumptions that it is possible to ever collect enough data to the point where no new possible themes, codes or ideas can be generated (Braun & Clarke, 2019). This approach does not align well with reflexive thematic analysis and was subsequently not used. Instead, we approached sample size using information power (Malterud et al., 2016); see *Methods>Participants and recruitment.*
**23.**	**Transcripts returned**	Were transcripts returned to participants for comment and/or correction?	Transcripts were not returned to participants, as this practice indicates that the role of the researcher is more passive, when they are equal participants in the generation of knowledge and data.
**Domain 3: Analysis and findings**			
**Data analysis**			
**24.**	**Number of data coders**	How many data coders coded the data?	One; data was coded by NH-S, with JB also familiarising herself with several transcripts; see *Methods>Data analysis*.

**25.**	**Description of the coding tree**	Did authors provide a description of the coding tree?	A coding tree was not used; a description of the process of reflexive thematic analysis is available in *Methods>Data analysis*.
**26.**	**Derivation of themes**	Were themes identified in advance or derived from the data?	Themes were inductive and data-driven; see *Methods>Data analysis* for further information.
**27.**	**Software**	What software, if applicable, was used to manage the data?	NVivo was used for data analysis.
**28.**	**Participant checking**	Did participants provide feedback on the findings?	Similar to item 23, this practice denies the active role of researchers in generating knowledge and data and suggests that there is a "correct" perspective, which does not align with our epistemological or ontological frameworks.
**Reporting**			
**29.**	**Quotations presented**	Were participant quotations presented to illustrate the themes / findings? Was each quotation identified?	Yes; see *Results*.
**30.**	**Data and findings consistent**	Was there consistency between the data presented and the findings?	As above.
**31.**	**Clarity of major themes**	Were major themes clearly presented in the findings?	As above.
**32.**	**Clarity of minor themes**	Is there a description of diverse cases or	As above.

	discussion of minor themes?	

Table S2. Additional Illustrative Quotes

Theme	Subtheme	Quotes
Theme 1. Helpful or harmful: Risk prediction as a "double-ended sword”	1a. Proactivity versus paranoia	“Could also be used to maybe raise more like awareness about the issues, so even if people were identified as low risk it might sort of help them sort of understand those conditions a bit further and just be more mindful of them should they ever like, come across someone with that mental health condition or they could develop it themselves.” (Elijah)<br><br>“If I was at high risk for diabetes, I'd like watch my diet, I would do the same if I was at like high risk for depression, I'd probably be thinking about it. So like maybe in terms of my routine, I'd be like, oh, it's really important to, like, go outside and just like, maximize time in the sun because I know that helps me, and maybe I would make more of an effort to do things that would almost like help prevent that developing.” (Deepa)<br><br>“I guess it's something that could trigger you to take action or try to check yourself.” (Myles)<br><br>"For people that don't know that they might be at risk, they can then put things in place to sort of lower that risk.” (Jessica)<br><br>“I think it would definitely maybe make me a bit more hypervigilant and hyper-aware than needed in terms of different areas of my life.” (Phoebe)

		"I think that could be difficult 'cause it could make you quite paranoid if you're just having a regular off day, you might start thinking to yourself, oh well, I am high risk because this [...] it could be quite easy to get yourself into a panic and like a spiral of being so worried about what the AI is predicted it could make you feel worse about yourself, which could then become a bit of a self-fulfilling prophecy where it flags all these people as being high risk and then they become high risk because they're so scared of it." (Violet)<br><br>"If you're kind of younger, I would say that there's some danger of kind of thinking, 'I'm high risk, so I already have these problems' and kind of placebo-ing them, if that makes sense, kind of causing yourself to have issues." (Robyn)<br><br>"It kind of gives off like a self-filling prophecy almost like if you know that you're at high risk perhaps you kind of internalise that and behave that way a bit more." (Noah)
	1b. Validation versus distress	"I think high could be (pause) validating, like if you are not doing well, they'll also have the concern of, 'oh well, you know a lot of people are doing a lot worse than me' or 'maybe I'm just making it up'. Then, 'oh no, that's-that seems to be-other people have looked at my like records and information also think there's a concern there, maybe I do have a valid concern'." (Max)

	"Maybe I would feel like a bit more secure in myself and feel like, okay, you know, things are going well." (Deepa)<br><br>"Maybe you'd worry less about, when you're, like, worried and anxious, you might feel like that's more normal and you don't need to worry about it." (Chloe)<br><br>"In certain situations for certain people [the models could be helpful], but then there are those people that I think it could actually stress them out too much and could worsen their mental health." (Hazel)<br><br>"It reinforces the idea that I could have anxiety, for example, and then, yeah, I guess it can create even more anxiety actually, kind of like a loop back effect." (Kabir)<br><br>"It is just again the stigma of everything, it could really 'cause you a lot of stress [...] people who are [at high-risk] feeling like that, like I don't know, it's ruining their sense of self and they're second guessing all their actions [...] and for people [at low-risk], they might think that their problems don't need to be listened to and no one needs to care about them because there are other people who have it worse. So I guess being labelled, regardless of what you're being labelled as, is going to affect you mentally in some negative ways." (Violet)

		"I don't think I want to live most of my adult life knowing that something might happen further down the line." (Poppy)
	1c. (En/dis)couraging support-seeking	"Prevention is better than cure [...] knowledge is a good thing so knowing that that's something that you're at risk of, I think getting that support would be really useful." (Jessica)<br><br>"I think being low risk might kind of encourage me to not pay as much attention to my mental health [...] I might kind of take more risks and end up putting myself in situations that aren't helpful for my mental health." (Alice)<br><br>"I actually don't think there's much benefit of being identified [as low risk] because it's almost like you might kind of start to feel like 'I can't be depressed'." (Noah)<br><br>"It might make people feel like less like they need to look after themselves if they do start having mental health problems, they might think that it's not something that they're likely to need help with because they've been labelled as low risk." (Mei)<br><br>"It has to have that wiggle room for people who may have been told their lower risk to still get maybe higher levels of support if they feel they need it." (Charlie)<br><br>"Just making sure that people understand that low risk doesn't mean that they're never going to get a

		mental health problem and just making sure that everyone is supported." (Robyn)
Theme 2. "Taken with a grain of salt": Are risk prediction models the way forward?	2a. Is it ethical?	"There's something kind of strange about the label of low risk because I think with a lot of mental health issues, the environmental factors are going to be very crucial to that, so I mean, if you're under a lot of stress or pressure later on, even though you might be labelled as low risk as a person, the environmental factors are not necessarily going to match up with that." (Mei)<br><br>"Just outright saying someone is high risk for depression with like - I don't know, just something about the way it sounds, sounds very like medical and very like formal. And I think that can be a bit much for some people." (Arif)<br><br>"I think sometimes being given a label that's like, 'you are high risk' can be like it can feel very definite […] I think even to it going like, 'you are currently high risk' would have a more positive effect because it's basically saying that-treating it as a fluid thing rather-based on circumstance rather than like feeling like an inherent thing." (Charlie)<br><br>I think it could be potentially dangerous to give a child a label like that […] maybe just not wording it as high or low risk like more wording as, 'we could tell that there's some stuff going on at home' or 'there may be something going on that could impact you in the future'. I think high or low risk is far too definitive and too boxed up." (Lucy)

		"I guess just like a bit more [...] clarity around how [risk scores are] calculated and that kind of thing, just to make it more like transparent, because [...] it seems like a little bit like scary." (Deepa)
	2b. Will it be accurate?	"I think mental health is quite a wide-ranging thing you know, as I've said, there's always context to things, especially related to mental health issues. And I don't think maybe a single questionnaire can kind of capture all that." (Kabir)<br><br>"I worry what a model could do in terms of there's so many human factors involved in developing mental health conditions, how far can a model go to predict that?" (Poppy)<br><br>"I think the they could end up being quite accurate, but there will always be people who slip through the cracks on both and there'll be people who get flagged incorrectly as having problems they don't and there'll be people who get flagged as being fine when they're not." (Violet)<br><br>"In mental health I feel like context is really important and unless the risk prediction is able to compensate and include every single part of context like, you know, adverse childhood experiences, like all of those different things, then it could, I don't know - maybe I'm a bit more pessimistic." (Deepa)<br><br>"Partially it would depend on the kind of data that goes into it, so some of the things you mentioned

	were like questionnaires, so that kind of depends on part of, yeah, like depends on the people giving accurate responses about themselves which I guess, depending on how subjective questions are, that could be difficult." (Mei)<br><br>"The person could just lie on the questionnaire if they like didn't feel comfortable sharing or something like that." (Arif)<br><br>"If they're being very, very honest with themselves [...] and just answer each questionnaire with like full truthfulness and honesty, then you can probably to be able to trust what the outcome of that risk prediction model is but if you're not very honest with yourself [...] I don't think you'd be able to trust what comes out of it." (Roshni)<br><br>"I think there's stuff you could definitely glean from that, but there's always things that like people won't mention unless explicitly asked or will still avoid anyway so it's never going to be incredibly accurate." (Max)<br><br>"We can't exactly be like, 'okay, give me every bit of your history' and then predicting it." (Kalani)<br><br>"I think there would just have to be like a very rigid and very comprehensive framework in place to see what data they'd be allowed to collect but [...] I don't know if it would make it as good of a risk prevention model if it's very limited." (Tara)

2c. Will it be secure?	"I'd probably have a couple of privacy concerns like how you're getting that data and stuff like that, but I think I'd be okay as long as it was anonymous." (Imogen)<br><br>"I guess it would depend how that data is collected like if you've opted in to be part of that or if it's sort of, you know, if you have no choice, especially if they're taking data off your phone so privacy could maybe be a concern." (Jessica)<br><br>"If that data's on my record how it's shared, like what does that mean for me like career-wise and is anyone going to - there have been so many hackings recently of like NHS data and like general government data- so yeah, maybe I'd be a bit like worried about that." (Deepa)<br><br>"The only big trustworthy issue would have been like using data from the phone app. However, if a person's already like consented to data being collected, if a person's already like understood that there will be privacy and confidentiality preserved by it then I don't have any big concerns I guess." (Andres)<br><br>"In terms of privacy of data, I think as long as it all stays within the same system, it's fine. Like the NHS collects data on all of us but I don't see any problem in that because it's for the NHS to provide us healthcare. But-and I trust the NHS isn't selling my data off to other organisations. I would feel less secure if I was just feeding my information into an

	app or something but didn't feel like it was part of an official government organisation that I'd grown up trusting. But I think as long as you know you're handling the data properly, I don't think it's a big issue." (Lucy)<br><br>"I also recognise there are like dozens of apps on my phone that have all this information about me that's readily shared and it's like outside the country [...] I think there's probably a lot more privacy laws in place with-I guess it would be medical information." (Jacob)<br><br>"Knowing that you can withdraw from it and withdraw the data so I think that would kind of make it a bit more trustworthy as well." (Phoebe)
2d. Is it feasible?	"Using the model to predict things could be a good way to get people with the right sort of support and the right level of support early on." (Mei)<br><br>"It's not going to be practical to give everyone personalised support so I guess that's just got to be a levelled thing of only if you're seen as the highest risk." (Lucy)<br><br>"One negative of maybe doing this as a prevention strategy is that someone might therefore do it for like a really, really long time and it might become tiring [...] like if it is something as intense as one-to-one, you can't just do that every week for years like that's just not feasible like in terms of like money, like NHS money, resources, but also the individual's

		time [...] I guess if it was really intense, then perhaps it would lead to like almost burnout of that intervention, like it becoming less effective and therefore maybe it wouldn't have as positive an impact over time.” (Poppy)
Theme 3. “It really depends on the person”: Respecting the individual in prevention	3a. Prevention is a personal choice	“I think choice is really important. I can imagine some people who might come up as high risk, but then might not want any support at all, and then others who might come up as low risk but might then want kind of, yeah, access to mental health apps to kind of maintain their mental health.” (Alice)<br><br>“I think that having opt-in style of, ‘here are your recommendations, but it's up to you to say whether or not you want that’, I think that's good.” (Andres)<br><br>“I think it probably is better to have a choice, but I feel like it's pretty important to encourage people to go.” (Chloe)<br><br>“I'd rather a consultation to see if it was something I wanted to be a part of, I don't think anything should be like forced onto someone, particularly if they're high risk of developing a condition but they don't actually have that condition.” (Poppy)<br><br>“The main one is don't overwhelm the person in one go. You know, give them stage by stage, don't bombard them all in one way, because that won't do any favours. Well, not for me it wouldn't anyway, I can't comment for other people, but you have to

	build kind of like a relationship with them. And then, yeah, hit them with more information, 'cause at the end of the day also the more comfortable they feel, the more information they'll give and the more valuable it will be, do you know what I mean?" (Theodore)<br><br>"I feel like it can only negatively impact you if you're not ready for it, or you're not prepared for it, or you're not wanting it and again, it's kind of shoved onto you, but obviously it should always be agreed to beforehand to prevent that issue from happening." (Kabir)<br><br>"I think either way the doctor tells me, 'This is the best course of action' I would take it. But yeah, obviously I wouldn't prefer someone making decisions for me." (Kalani)
3b. Individual differences impact personalisation	"I think particularly like the counselling, it enables you to think about like early warning signs of depression and anxiety and that could prevent someone developing kind of a full-on episode if they were able to kind of catch it early." (Alice)<br><br>"The thing is with counselling, the client receiving the counselling has to be like engaged and want to be there and I don't necessarily believe that most or that other people would do that, especially if they're not experiencing the symptoms yet if it's just to prevent it." (Hazel)

	"Group work could be good for some people like you said 'cause it allows people to like kind of connect with other people who might have similar issues." (Arif)<br><br>"For maybe lower risk people just maybe like a list of tips would be good, again, access to apps I think would be good for both high and low risk people just because maintaining positive mental health I think is a good thing across the board. But maybe more specific or targeted apps and resources for those that are high risk will be good." (Elijah)<br><br>"I think it could be helpful to get the right level of support for how [...] you're not put in like too high or low intervention for your needs." (Chloe)<br><br>"It's like you go to a pharmacy, you have a fever, they'll give you paracetamol, but obviously the dose has to be tailored according to your weight and height the same way it needs to be tailored." (Kalani)<br><br>"I think personalised intervention is very important 'cause it's tailored to that person and some specific treatments or interventions might not necessarily work for some, but it might work for others [...] it's ensuring that people have access to the resources that feel work for them, or that a mental health professional thinks would be most effective for that person." (Elijah)

		"If you personalise it to how each person is feeling and how each person goes about in their daily life, then I feel like it would be much more better in helping them out, because if you just create a general intervention, not every person is going to be able to fit it [...] there's a lot of factors - age, culture, gender, or different things that could affect how the person goes through or manages their anxiety and depression so making one for all is not the best idea." (Roshni)
Theme 4. "You still need like a person in the process": The importance of human involvement	4a. Involvement in development	"I think definitely just listening to a broad range of people, perhaps, and just getting their opinions and advice on it." (Elijah)<br><br>"You also have to be able to educate them on it so that it's not just, 'you are at risk, here are the things you need to do', but here's at risk, here's why and why it matters from your-from what you've given us." (Andres)<br><br>"I think the only concern I would have is like I don't understand the model enough [...] I would want to develop a deep understanding of like how this has been formulated, with what information have I been labelled high or low risk, and it, like, is there context here?" (Deepa)<br><br>"More information about what the risk prediction, like, modelling is based on, so why it's come to that conclusion of, you know, high or low risk, and what the sort of calculations are behind it might [...] make me a bit more comfortable and give me a bit more

		insight into how that's come to that conclusion." (Elijah)<br><br>"I suppose like education about why these things, these sessions or whatever it is, are potentially going to be beneficial and what kind of positive outcomes they could create [...] because then I'd be more encouraged to want to kind of turn up and engage in the sessions." (Hazel)
	4b. Involvement in implementation	"I think they can be useful but you still need like a person in the process, actually like talking and getting the information. There might be more nuanced than an algorithm or something can process." (Charlie)<br><br>"If the practitioner has like an understanding of previous support that that individual might have had and what did and what didn't work, I think that would probably be useful towards the intervention." (Poppy)<br><br>"I feel like breaking the news in person would definitely be a bit more easier and better to do in case like some sort of sensitive information needs to be discussed as well." (Roshni)<br><br>"Just having that kind of medical professional tell you as opposed to effectively a computer. Just keeps things, yeah, kind of proper." (Robyn)<br><br>"Knowing these people kind of are like properly trained and [...] it is going to really help. Maybe if I

	know the intervention probably is going to help, I think it would make you feel more comfortable, I think I'd feel more comfortable." (Chloe)

References

Berger, R. (2015). Now I see it, now I don't: Researcher's position and reflexivity in qualitative research. *Qualitative Research, 15*(2), 219-234. https://doi.org/10.1177/1468794112468475

Braun, V., & Clarke, V. (2021). To saturate or not to saturate? Questioning data saturation as a useful concept for thematic analysis and sample-size rationales. *Qualitative Research in Sport, Exercise and Health, 13*(2), 201-216. https://doi.org/10.1080/2159676X.2019.1704846

Braun, V., & Clarke, V. (2021). *Thematic Analysis: A Practical Guide*. SAGE Publications Ltd.

Malterud, K., Siersma, V. D., & Guassora, A. D. (2016). Sample size in qualitative interview studies: guided by information power. *Qualitative Health Research, 26*(13), 1753-1760. https://doi.org/10.1177/1049732315617444

Tong, A., Sainsbury, P., & Craig, J. (2007). Consolidated criteria for reporting qualitative research (COREQ): a 32-item checklist for interviews and focus groups. *International Journal for Quality in Health Care, 19*(6), 349-357. https://doi.org/10.1093/intqhc/mzm042
